# The Mediation Effect of Health Literacy on Social Support with Exchange and Depression in Community-Dwelling Middle-Aged and Older People in Taiwan

**DOI:** 10.3390/healthcare9121757

**Published:** 2021-12-19

**Authors:** Ya-Ling Shih, Chia-Jung Hsieh, Ya-Ting Lin, Yi-Zhu Wang, Chieh-Yu Liu

**Affiliations:** 1 School of Nursing, College of Nursing, National Taipei University of Nursing and Health Sciences, Taipei 112303, Taiwan; tiffanysyl0902@gmail.com (Y.-L.S.); yatinglin@smc.edu.tw (Y.-T.L.); s10301002@cjc.edu.tw (Y.-Z.W.); 2 ST. Mary’s Junior College of Medicine, Nursing and Management, Yilan 266006, Taiwan; 3 Department of Speech Language Pathology and Audiology, National Taipei University of Nursing and Health Sciences, Taipei 112303, Taiwan; chiehyu@ntunhs.edu.tw

**Keywords:** health literacy, social support with exchange, depression, mediation effects, middle-aged and older people

## Abstract

The proportion of the world’s population that are over 60 years old is increasing rapidly. The physical and mental health of older people is affected by depression. Health literacy is a major determinant of health and healthcare for the aging; middle-aged and older people with high health literacy are more likely to maintain a healthy lifestyle, and control or manage their chronic diseases. Therefore, this study explored the relationship between health literacy, social support with exchange, and depression, in middle-aged and older adults in the community, using data from the 2015 Taiwan Longitudinal Study on Aging (TLSA) database. Of the 7636 participants, 1481 (19.4%) were middle-aged or older persons with depression symptoms. We found age, gender, and education level to be significantly related to health literacy status, social support with exchange, and depression. Health literacy was positively correlated with depression and social exchange in social support with exchange, whereas the emotional support component of social support with exchange was negatively correlated with depression. Regression-based process analysis was used to verify the mediation effect of health literacy. Our results indicated that when health literacy was entered into the regression model (*a* × *b* path), the effect of social exchange on depression was insignificant (*c*′ = −0.01, *p* = 0.84), indicating a complete mediation effect. These findings suggest that improving health literacy may offset the impact of social support with exchange on depression, and lead to the mitigation of depression in middle-aged and older people in Taiwanese communities.

## 1. Introduction

The aging process is very complicated, with obvious changes both physically and psychologically. As people get older, the risk of having multiple coexisting chronic diseases increases. Recently, 92 diseases have been identified as age related, accounting for 51.3% (95% UI 48.5–53.9) of all global burden among adults in 2017 [1]. However, physical degradation, chronic diseases, and disability associated with aging may cause psychological pressure, negative emotional feelings, and even depression [2]. This is a phenomenon that requires attention in an aging society [2]. The World Health Organization (WHO) reported that the incidence of unipolar depression is 7% in the elderly, and accounts for 5.7% of years lived with disability (YLDs) for those over 60 years old [3]. Depression can cause great pain and adversely affect the activities of daily living, even more so than other chronic diseases often associated with a profound impact on (dis)ability.

In a systematic review on the morbidity rate of depression in older adults, 74 studies involving a total of 487,275 people aged 60 years and above were included. The morbidity rate of depression was determined to be 4.7–16.0%, with a median morbidity rate of 10.3% [4]. Depression is associated with physical and psychological problems. Soysal et al. [5] found that, among 2167 older patients with depression, the prevalence of frailty was 40.40% (95% CI 27.00–55.30, I^2^ = 97%). Moreover, depression may lead to suicide. The Taiwan Ministry of Health and Welfare reports [6] indicate that the suicide rate among middle-aged and older people was the highest among all age groups (24.7%), and depression was an important factor of this [7].

Reviews of published literature indicate that many factors are associated with depression. Social support is one such factor found to be associated with depression [8]. Moreover, high rates of low health literacy among older adults, along with a high prevalence of chronic conditions may lead to increased levels of depression symptoms [9,10]. However, the relationship between the level of health literacy, social support with exchange, and depression remains largely unknown and unexplored. Thus, the present study investigates the mediation effect of health literacy on the relationship between social support with exchange and depression.

### 1.1. Social Support with Exchange and Depression

Social support is a mechanism that relieves life pressure and promotes health at the same time, thereby contributing to positive psychological effects. Middle-aged and older people with less social support have a higher incidence of depression [8,9,11,12,13,14,15,16]. A systematic review of 24 studies found that good social support is associated with the reduction in depression [17]. However, relying on the support of others may lead to guilt and anxiety [18]. In contrast, if older adults are provided with instrumental assistance, it can help prevent a decline in their daily living activities [19,20]. Brown et al. [21] also identified that older adults who were provided tools by friends, relatives and neighbors exhibited significantly reduced mortality. There is accruing evidence that providing social support may be more beneficial than obtaining social support [21,22]. Thus, our first hypothesis is that social support and exchange affects depression (Hypothesis 1). The nature of such effect forms one of the questions addressed in the present study.

### 1.2. Social Support with Exchange and Health Literacy

Health literacy is an important factor in determining public and personal health, and is regarded as the core of patient-centered care [23]. Nutbeam [24] defined health literacy as a personal, cognitive and social skill that determines the individual’s ability to obtain, understand, and use information to promote and maintain good health. Poor health literacy is a silent epidemic across the globe, affecting every aspect of health [25]. In several reports, a lack of health literacy has been associated with higher mortality, poor self-management skills, lower satisfaction with medical communication, poor awareness of diseases, higher hospitalization and emergency medical-use rates, incorrect use of medication, low utilization of preventive healthcare services (such as screening), high prevalence of chronic diseases (such as cardiovascular disease, diabetes, and obesity, etc.), and high healthcare costs [26,27].

There are suggested associations between health literacy and social support. This includes reports by Liu et al. [28], showing that health literacy was positively correlated with social support (*β* = 0.151, 95% CI: 0.077, 0.224), but negatively correlated with depression (*β* = −0.173, 95% CI: −0.246, −0.1) in 637 adults aged ≥65 years with hypertension and diabetes, living in the community. This indicates that older people with higher health literacy tend to have better social support and relatively lower levels of depression. On this premise, we next hypothesized that the interaction of social support with exchange is affected by health literacy (Hypothesis 2), and investigated the nature of the probable effect of health literacy on social support.

### 1.3. Health Literacy and Depression

Many studies have shown that poor health literacy is significantly associated with increased incidences of depression [9,10,15,29,30,31,32], including middle-aged and older adults. A study of 3260 older people showed that, compared with persons with sufficient health literacy skills, those with insufficient health literacy were 1.2 times more likely to be depressed (95% CI: 0.9–1.7). However, this was mostly explained by the bidirectional relationship between health literacy and depression, which may be mediated by health status [9]. Hsu et al. found that improving the health literacy of diabetic women elicited reduced psychological distress in those with depression, as shown by the negative correlation between health literacy and depressive tendencies [10]. In fact, for each 1-point rise in the Chinese Health Literacy Scale for Diabetes score, the Center for Epidemiologic Studies Depression Scale (CES-D) score decreased by 0.17 points (*z* = −2.05, *p* = 0.042) [10]. Thus, extrapolating from the general population to a more specific age group, the present study evaluated the relationship between health literacy and depression in middle-aged and older adults, with the working hypothesis that health literacy is inversely associated with depression (Hypothesis 3).

### 1.4. Health Literacy as a Mediator between Social Support and Depression

This present study investigates the relationship between health literacy, social support with exchange, and depression. Liu et al. [28] described health literacy as a predictor of social support and depression. Zhang et al. [32] concluded that physical comorbidity and health literacy mediate the relationship between social support and depression among patients with hypertension in their study of 549 hypertensive adults (95% CI: −0.282 to −0.097). However, little is known about the protective effect of health literacy on middle-aged and older people. Against this background, we further hypothesized that health literacy has a mediation effect on the relationship between social support with exchange and depression (Hypothesis 4).

To further clarify the mediation effect of health literacy on the relationship between social support with exchange and depression, this study also adjusted other related factors. As far as we know, this is the first study to investigate the mediation effect of health literacy on social support with exchange and depression in middle-aged and older people in Taiwan.

### 1.5. Theoretical Framework

According to Sørensen, et al. [33], health literacy is linked to literacy and entails people’s knowledge, motivation and competency to access, understand, appraise, and apply health information towards making judgments and decisions in everyday life concerning healthcare, disease prevention, and health promotion, to maintain or improve quality of life during one’s life course. Sørensen, et al. [33] proposed an integrated model of health literacy which combines the qualities of a conceptual model, outlining the main dimensions of health literacy, and those of a logical model, showing the proximal and distal factors which impact health literacy, as well as the pathways linking health literacy to health outcomes. The core of the model is the ability to acquire, understand, evaluate, and apply health-related information across the three dimensions of health literacy: namely, health care, health promotion and disease prevention. In addition to the components of health literacy, the model also shows the main antecedents and consequences. The antecedents are personal determinants (e.g., age, gender, race, socioeconomic status, education, occupation, employment, income, literacy) and situational determinants (e.g., social support, family and peer influences, media use and physical environment). Moreover, health literacy could affect health service use, health costs, heath behavior and health outcomes at the individual level and participation, empowerment, equity and sustainability at the population level.

We used this model as the framework of this study. In summary, the path model of this study aimed to confirm the mediation effects of health literacy on the relationship between a situational determinant, namely, social exchange with emotional support, and a health outcome, depression, with the personal determinants of middle-aged and older people as control variables.

## 2. Methods

### 2.1. Study Design and Data Collection

Since 1987, Taiwan has been conducting surveys and studies collectively known as the “Taiwan Longitudinal Study on Aging” (TLSA), in order to understand the health and living conditions of middle-aged and older people over the age of 50. Eight research sessions were completed between 1989 and 2015 [34]. The TLSA survey adopts stratified random sampling, allowing the collected data to fully reflect the physical, psychological and social aspects of the participants. Data and results from the TLSA project can also serve as empirical basis for the formulation of social and healthcare policies for older adults. This study used data from a component of the TLSA termed ‘the Long-term Tracking Survey of the Physical and Mental and Social Life of the Middle-aged and Older people in Taiwan in 2015’.

### 2.2. Participants

The total number of participants with complete data was 7636. The computed required number of participants was 1073, based on an effect size |ρ| = 0.1, error probability (α) = 0.05, and power (1-β err prob) = 0.95. Surpassing the required number, of the 7636 participants, 1481 (19.4%) were middle-aged and older persons with depression symptoms.

Exclusion criteria: Of the total 7636 enrolled subjects, 6155 were excluded because they had depression symptoms index scores 0–8, had no documented history of depression, and were younger than 50 years old.

Inclusion criteria: In all, 1481 middle-aged and older people (19.4%) had depression symptoms index scores ≥ 9, were classified as having depression symptoms, and thus considered eligible for further comparative studies.

All personally identifiable information from the TLSA are encrypted to protect the participants. This study was approved by Fu Jen Catholic University (FJU-IRB No: C109147), and conducted following the Declaration of Helsinki guidelines on research involving human subjects.

### 2.3. Measures

#### 2.3.1. Demographic Characteristics

The demographic characteristics evaluated included gender, age, and education level. Consistent with Lin et al. [35], according to their age, participants were divided into two groups: 50 to 64 years, and 65 to 85 years. Similar to the levels of education used by Xu et al. [36], the study cohort was also divided into two strata: “junior high school or lower” and “senior high school or above”.

#### 2.3.2. Social Support with Exchange Scale

This 7-item scale consisted of two sub-scales: social exchange and emotional support. The three questions on social exchange were scored on a scale of (0) No, (1) Occasionally, and (2) Often. The total score range of the three items was 0–6. A higher total score indicated a greater frequency of social exchange, which in turn implied better social support. There were four questions on emotional support. The scoring method was divided into (1) Very supported (2) Supported (3) Normal (4) Not supported (5) Very unsupported. The total score range of the four items was 4–20. A lower total score indicated better emotional support of the respondent. In this study, the Cronbach’s α of the scale was 0.70. The social support with exchange scale facilitated the understanding of the social status of the middle-aged and elderly in Taiwan, such as the participant’s “family structure, living arrangements, social support, leisure activity, socioeconomic status, life satisfaction, occupation and retirement, and awareness and utilization of services provided by the government” [37].

#### 2.3.3. Health Literacy Scale

The scale contained three dimensions: health care, health promotion, and disease prevention. There were a total of 9 items, which were scored on a 5-point Likert-type scale. The total score range was 9–45. A score of 20 points or less was considered to indicate good health literacy. In this study, the Cronbach’s α of the scale was 0.86. The TLSA health literacy scale is a reliable and valid instrument for measuring health literacy in middle-aged and older people [38]. As alluded to already, the health literacy scale allows the comparison of the “differences in health and social status among subgroups of people characterized by their socio-economic background” [37].

#### 2.3.4. Center for Epidemiological Studies Depression Scale (CES-D)

The abbreviated version of the Center for Epidemiological Studies Depression Scale (CES-D Scale) was used to measure depression during the survey. The scale was composed of 11 questions out of the original 20 questions on the CES-D developed by Radloff [39]. The short version of the CES-D scale included three factors: physical symptoms, depressive emotions and positive emotions. Each of these 11 items was scored on a 4-point Likert-type scale of “rarely (<1 day) = 0” to “frequently or consistently (more than 4 days) = 3”. The total score range was 0–33. A higher score indicated a higher frequency of depression, whereas a score of 8 or higher indicated obvious symptoms of depression. The Cronbach’s α ranged from 0.76 to 0.81, indicating that the TLSA short version of the CES-D scale, which was based on the Iowa EPESE (estabilished populations for epidemiologic study of the elderly), had good internal consistency reliability [40]. In this study, the Cronbach’s α of the scale was 0.71. 

### 2.4. Statistical Analysis

In this study, the Chinese version of SPSS 22.0 (SPSS Inc., IBM Corp., Chicago, IL, USA) was used for data analysis. All variables adopted descriptive statistics (such as the median and standard deviation of continuous variables, the percentage of categorical variables). Variable correlation analysis and Univariate linear regression were used to examine the association between social support with exchange, health literacy, covariance, and depression scales.

The process procedure for SPSS Version 3.5.2 statistical software Model 4 (Hayes Process macro for SPSS) [41] was used to analyze the mediation effect of health literacy on social support with exchange and depression in middle-aged and older adults. The bootstrap participants numbered 5000, and a 95% confidence interval was set to achieve sufficient statistical analysis power. Since the gender, age, education level and key variables were all related, we used these three as covariates in the mediation regression model.

## 3. Results

### 3.1. Descriptive Statistics

The descriptive statistics of the participants are listed in Table 1. Our total participants consisted of 3917 females (51.3%) and 3719 males (48.7%). The largest groups were those 65–85 years old and above (52.4%) and those with a primary or junior high school level education (66.9%). The average depression score was 4.3 ± 5.3 (range, 0–32).

The depression group was older and mostly female, and had a low education level. Social support with exchange–social exchange was low, social support with exchange–emotional support was poor, and health literacy was also low. In addition, the average scores of social support with exchange–social exchange, social support with exchange–emotional support, and health literacy were 2.03 ± 0.01, 7.33 ± 2.55, and 15.12 ± 5.92, respectively. Most (79.2%) of the participants had sufficient health literacy.

### 3.2. The Correlation of Health Literacy and Social Support with Depression

The correlations between the key variables are listed in Table 2. Health literacy was positively correlated with depression (*r* = 0.354, *p* < 0.001), indicating that better health literacy was related to lower depression. Social support with exchange–social exchange was negatively correlated with depression (*r* = −0.145, *p* < 0.001), indicating that better social exchange was associated with a lower degree of depression. Social support with exchange–emotional support was positively correlated with depression (*r* = 0.377, *p* < 0.001), suggesting an association between worse emotional support and a higher degree of depression.

### 3.3. The Mediation Effect of Health Literacy

**Figure 1:**
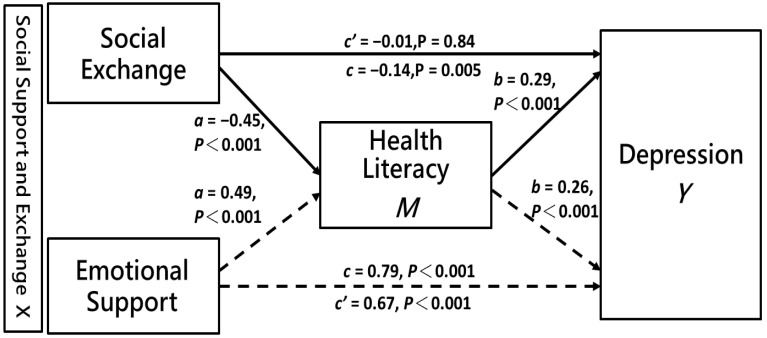
Mediation effect of health literacy on the relationship between social support with exchange and depression.

Based on Baron and Kenny [42], this study proposes the conditions and tests of the intermediary variables. The results of the model are shown in Figure 1. Paths *a*, *b*, and *c* represent standardized regression coefficients between paths. Path *c* presents the association between social support with exchange and depression, path *a* shows the association between social support with exchange and health literacy, path *b* shows the association between health literacy and depression, and path *c’* presents the mediation effect of health literacy on social support with exchange and depression.

The direct, indirect and total effects of the key research variables are presented in Table 3. The indirect effect (*ab*) was defined as the product of coefficients *a* and *b* [43]. If the 95% bootstrap CI did not contain zero, the indirect effect was considered to be significant, indicating that there was a mediation effect. Health literacy had a mediation effect on social support with exchange and depression (*ab* = −0.13, 95% CI = −0.17 to −0.010; *ab* = 0.13, 95% CI = 0.11 to 0.15).

The total effects of social support with exchange–social exchange and emotional support on depression in middle-aged and older people were significant (*c* = −0.14, *p* = 0.005; *c* = 0.79, *p* < 0.001), which supported Hypothesis 1. The effects of social support with exchange–social exchange and emotional support on the health literacy of middle-aged and older people respectively were significant (*a* = −0.45, *p* < 0.001; *a* = 0.49, *p* < 0.001), thus supporting Hypothesis 2. The effect of health literacy on depression was (*b* = 0.29, *p* < 0.001), which supported Hypothesis 3.

**Figure 2:**
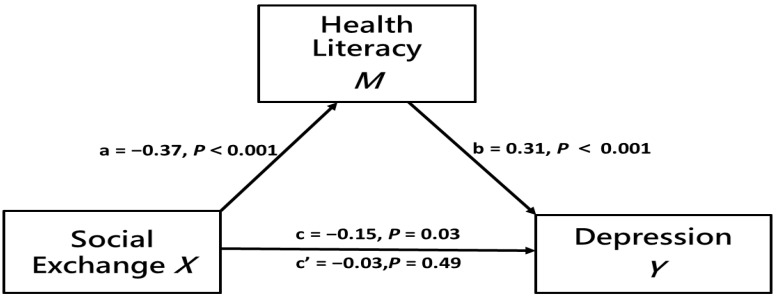
Mediation effect of health literacy on the relationship between social support with exchange–social exchange and depression after adjustment for gender, age and education.

When health literacy was incorporated into the regression (*a* × *b* path), the effect of social support with exchange–social exchange on depression became non-significant (*c*′ = 0.01, *p* = 0.84), indicating that health literacy could completely mediate depression in social support with exchange–social exchange. Figure 2 shows that, after adjustments for gender, age, and education level, health literacy still had a complete mediation effect on the relationship between social support with exchange–social exchange and depression. In addition, the influence of social support with exchange–emotional support on depression was significant (*c′* = 0.67, *p* < 0.001), indicating that health literacy could have a partial mediation effect on depression between social support with exchange–emotional support, accounting for 16.46% of the total effect. These results supported Hypothesis 4.

## 4. Discussion

This section will discuss the hypothesis proposed in this research and report the related limitations.

### 4.1. Health Literacy Status Positively Correlates with Social Support with Exchange, but Is Inversely Associated with Depression

In this nationally representative study, with middle-aged and elderly community-resident participants, we found that the prevalence of depression in middle-aged and older people was 19.4%, which was lower than the prevalence reported in the previous study (35.2%) [44] and lower than those in India (41%) and South Asian countries (42%) [45]. However, it was higher than the world’s median of depression in an older population of 10.3% (interquartile range [IQR], 4.7–16.0%) [4], which in turn was higher than the 13% rate in the United States [46], 13.9% in Sri Lanka [13] and 18.5% in Thailand [47]. Therefore, surveys of depression in older adults are easily affected by the differences in customs and cultural backgrounds of various countries.

This study found that, in terms of gender differences, middle-aged and older females were more likely than their male counterparts to have depression. This finding was consistent with those in previous studies that the female gender is a risk factor of depression in old age [2,13,44,45,46,47,48,49,50,51,52]. Moreover, age is positively correlated with depression, as age is an important determinant of mental health. Due to the normal aging of the brain, the deterioration of physical health, and brain diseases, the overall prevalence of mental and behavioral disorders has been shown to increase with age [2,45]. We demonstrated that the severity of depression increases with age, as was consistent with the findings of previous studies [2,45,48,49]. Furthermore, we found that a low educational level was significantly associated with a higher risk of depression, corroborating the findings of Portellano-Ortiz et al. [50] and Ylli et al. [52]. In a cross-sectional study, a total of 93,590 people over 55 years of age in 18 countries completed questions related to depressive symptoms using the shortened CES-D or EURO-D scale. The study indicated depression prevalence was generally highest among women, individuals aged 75 years or older, those who were divorced, widowed, or single, and those who did not attain a secondary education [53], which is similar to our research results.

Our data showed that 79.2% of the middle-aged and elderly people had sufficient health literacy, similar to the results of a Finnish study, which found that 51.4% had sufficient literacy and 12.3% had excellent health literacy [54]. However, this is higher than the figures reported in studies from the United Kingdom, the United States, Taiwan, and Germany, with 49.5%, 51%, 53.7%, and 66–80% of persons older than 65 years having poor or limited health literacy, respectively [55,56,57,58], or worse still in Turkey, where 85.1% of the elderly are considered to have “problematic or insufficient” health literacy [59].

### 4.2. Components of Social Support with Exchange Differentially Affect Depression

Social support elicits better mental health. The results of this study indicate a negative correlation between social support with exchange–social exchange and depression. A higher score for “social exchange” indicated that better social exchange was associated with lower levels of depression. This may be related to the traditional concept in Chinese culture that it is more blessed to give than to receive. In Chinese society, providing support to others increases happiness in older people [60]. Brown et al. [21] pointed out that giving support may be an important part of interpersonal relationships, and has considerable value for health and well-being. Chen et al. [61] stated that encouraging individuals to provide appropriate support, such as helping others, and being willing to accept support may be beneficial for well-being and longevity. Conversely, social support with exchange–emotional support was positively correlated with depression, implying that a higher score on emotional support indicated worse emotional support, and thus a stronger likelihood to depression. This impact of social support on depression is consistent with findings from other studies [9,13,62,63].

Gyasi et al. [64] found meaningful social support to be a key element of life in older people. By strengthening opportunities to establish closer interpersonal relationships with others, older adults could improve their mental health, independence and quality of life. Therefore, social support with exchange helps people release negative psychological pressure during the aging process.

### 4.3. Social Support with Exchange Reflects Health Literacy Status

Social support with exchange–social exchange was negatively correlated with the health literacy of middle-aged and older people, which means that better social exchange was associated with higher health literacy (a lower score). Consistent with the conclusions of previous studies [28,58,65,66,67], we found emotional support was positively correlated with health literacy, suggesting that if the degree of support was poor, the level of health literacy was also low. Thus, the rationality of De Wit et al.’s proposed practice of collaborative learning and social support to improve the health literacy of older people [68]. Moreover, social support could help alleviate the negative effects of low health literacy [65].

### 4.4. Inadequate Health Literacy Is Implicated in Mental Health Deterioration and Depression

This study found that health literacy was positively correlated with depression. Inadequate health literacy in middle-aged and older people was associated with the deterioration of physical and mental health, including increased depression. This finding aligns with those previously reported [9,10,31,54], and therefore it can be stated that insufficient health literacy is strongly related to depression. Gazmararian’s team in their seminal study of 3260 elderly people found that individuals with inadequate health literacy were 2.7 times (95% CI, 2.2–3.4) more likely to be depressed, compared with individuals with adequate health literacy [9]. In addition, Do et al. [69] in their study of 928 adults aged 60–85, reported that every one-point increase in health literacy decreased the likelihood of depression by 9% (OR, 0.90; 95% CI, 0.87, 0.94; *p* < 0.001). Concurring with Parikh et al. [70], we found that people with low health literacy often feel shame and embarrassment, which can cause social isolation and constitute a serious psychological barrier to seeking help. This, in part, explained the observed higher odds of depression among people with lower health literacy than their peers with higher health literacy. Thus, we posit that improving health literacy has a health protective effect for older people, and is a protective factor against depression.

For contextualization, the current COVID-19 pandemic is associated with increased anxiety and mental health problems in the public [71,72,73] and, as established by Robb et al., ~13% of the elderly feel worse in terms of depression [51]. However, a 4–5% reduction in the likelihood of depression was reported for each unit increase in health literacy score, further highlighting the protective effect of health literacy against depression during the pandemic [74].

### 4.5. Health Literacy Significantly Mediates the Relationship between Social Support with Exchange and Depression

To the best of our knowledge, this study is the first to analyze the relationships between social support with exchange, health literacy and depression among the elderly in the community. Our results showed that the health literacy status of middle-aged and older people has a mediation effect on the relationship between social support with exchange and depression.

When social support with exchange and health literacy were input into the final mediation model, the association between social exchange and depression was eliminated (complete mediation), and the relationship between emotional support and depression was reduced (partial mediation), accounting for 16.46% of the total effect size. The reason for this may be that the need for listening and caring is extremely important in Chinese culture and society [63]. Our findings on the mediation effect of health literacy is consistent with those by Zhang et al. [32] and Zou et al. [15], validating the working hypothesis of the present study. Health literacy mediates the impact of social support with exchange on depression among the elderly in the community. Therefore, the improvement of health literacy and intervention measures require due attention. Duong et al. noted that entertainment series and educational TV programs on health promotion and health-related community activities help increase health knowledge and health behaviors, thereby improving health literacy [75]. As rightly opined by Nutbeam [24], findings in the presented study do suggest that the improvement of health literacy should emphasize more personal communication, community-based education outreach and health education content, with a focus on suitable equipment to overcome structural barriers to people’s health. Therefore, we recommend that coping strategies and support resources be provided to help middle-aged and older people improve their health literacy and mitigate or prevent depression.

**Figure 3:**
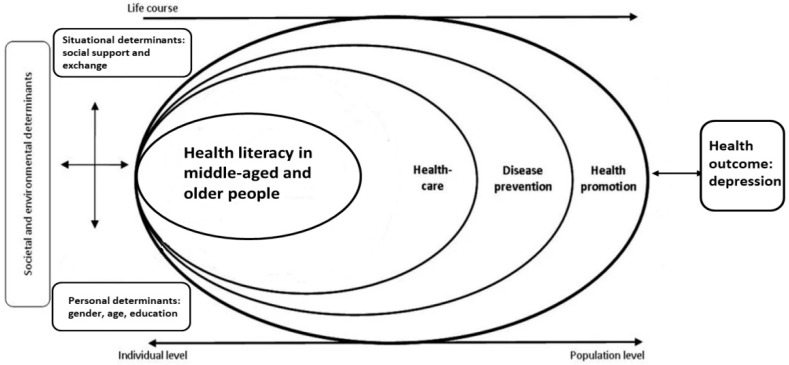
A simplified model for integrated model of health literacy and key variables in research.

Herein, our results (Figure 3) echo the health literacy integration model proposed by Sørensen et al. [33]. This study confirmed the mediation effects of health literacy on the relationship between situational determinants (social support with exchange), personal determinants (age, gender, and education level) and the health outcomes (depression) of middle-aged and older people.

### 4.6. Limitations of This Study

This study has some limitations. First, the individuals in this study were middle-aged and older people living in the community. Compared with individuals living in institutions, they may have relatively better mental health, social support with exchange, and health literacy. Second, because the health literacy assessment was a component of the first survey in 2015, continuous data analysis could not be done. Third, unlike analytic devices used for clinical diagnosis, the CES-D indexes only self-report current symptoms. Therefore, depression symptoms may be overestimated. Finally, the inclusion of other age groups in the study participants may also be necessary in extending the conclusions to a larger population.

## 5. Conclusions

In middle-aged and older people, social support with exchange differentially affects depression, and this association is mediated by health literacy status. Improving health literacy offsets the adverse effects of social support with exchange on depression. In view of these results, multidisciplinary intervention measures should be formulated to increase the social exchange component of social support with exchange and improve health literacy, so as to reduce the likelihood and incidence of depression. In addition, the results of this study echo Sørensen’s health literacy integration model, which extends the need to improve health literacy and patient/family engagement, empower people to take charge of their health and better prepare them to deal with health crises, rather than becoming passive recipients of services. This is an integrated people-oriented health service. The results reported herein may serve as an evidence-based reference for evaluating and/or mitigating depression in middle-aged and older people in Taiwan.

## Figures and Tables

**Table 1:** Comparison of participant characteristics between non-depressive groups and depressive groups.

Variables	Total		Depression Status				*p*
			Non-Depression		Depression		
	*n* (mean)	% (S.D)	*n* (mean)	% (S.D)	*n* (mean)	% (S.D)	
Age	7636		6155	80.60	1481	19.40	<0.001
50–64	3633	47.60	3097	50.30	536	36.20	
65–85+	4003	52.40	3058	49.70	945	63.80	
Gender	7636		6155	80.60	1481	19.40	<0.001
Male	3719	48.70	3146	51.10	573	38.70	
Female	3917	51.30	3009	48.90	908	61.30	
Education level	7636		6155	80.60	1481	19.40	<0.001
Junior high school or below	5112	66.90	3946	64.10	1166	78.70	
Senior high school or or above	2524	33.10	2209	35.90	315	21.30	
Social support with exchange							
Social exchange	(2.03)	(0.01)	(2.23)	(0.73)	(2.02)	(0.85)	<0.001
Emotional support	(7.33)	(2.55)	(6.96)	(2.24)	(8.82)	(3.13)	<0.001
Health literacy	(15.12)	(5.92)	(14.27)	(0.05)	(18.57)	(6.71)	<0.001

Data are presented as n (%) or (mean ± standard deviation).

**Table 2:** The correlation among key variables.

	Social Exchange	Emotional Support	Health Literacy	Depression
Social exchange				
Emotional support	−0.239 **			
Health literacy	−0.102 **	0.212 **		
depression	−0.145 **	0.377 **	0.354 **	

Abbreviations: ** *p* < 0.001.

**Table 3:** Models of the mediating role of health literacy in the relationship between social support with exchange and depression.

	Independent Variable	Mediator	Dependent Variables	Effect of *X* on *M*	Effect of *M* on *Y*	Direct Effect	Indirect Effect	Total Effect
	*X*	*M*	*Y*	*a*	*b*	*c*′	(*a* × *b*) 95% CI	*c* = *c*′ + *a* × *b*
Model 1	Social Exchange	Health Literacy	Depression	−0.45	0.29	−0.01	−0.13	−0.14
				(SE = 0.06) ***	(SE = 0.01) ***	(SE = 0.05)	(−0.17~−0.10)	(SE = 0.05) **
	Emotional Support			0.49	0.26	0.67	0.13	0.79
				(SE = 0.03) ***	(SE = 0.01) ***	(SE = 0.02) ***	(0.11~0.15)	(SE = 0.02) ***
Model 2	Social Exchange	Health Literacy	Depression	−0.37	0.31	−0.03	−0.11	−0.15
				(SE = 0.05) ***	(SE = 0.01) ***	(SE = 0.05)	(−0.14 −0.09)	(SE = 0.05) **

Model 1: unadjusted; Model 2: adjusted gender, age and education. ** *p* < 0.01; *** *p* < 0.001.

## Data Availability

The data that support the findings of this study are available from Health Data Science Center, Taiwan but restrictions apply to the availability of these data, which were used under license for the current study, and so are not publicly available. Data are however available from the corresponding author upon reasonable request and with permission of the Taiwan Ministry of Health and Welfare.
